# Image Classification Based on Light Convolutional Neural Network Using Pulse Couple Neural Network

**DOI:** 10.1155/2023/7371907

**Published:** 2023-03-14

**Authors:** Maminiaina Alphonse Rafidison, Hajasoa Malalatiana Ramafiarisona, Paul Auguste Randriamitantsoa, Sabine Harisoa Jacques Rafanantenana, Faniriharisoa Maxime Rajaonarison Toky, Lovasoa Patrick Rakotondrazaka, Andry Harivony Rakotomihamina

**Affiliations:** Telecommunication-Automatic-Signal-Image-Research, Laboratory/Doctoral School in Science and Technology of Engineering and Innovation/University of Antananarivo, Antananarivo 101, Madagascar

## Abstract

Recently, most image classification studies solicit the intervention of convolutional neural networks because these DL-based classification methods generally outperform other methodologies with higher accuracy. However, this type of deep learning networks require many parameters and have a complex structure with multiple convolutional and pooling layers depending on the objective. These layers compute a large volume of data and it may impact the processing time and the performance. Therefore, this paper proposes a new method of image classification based on the light convolutional neural network. It consists of replacing the feature extraction layers of standard convolutional neural network with a single pulse coupled neural network by introducing the notion of foveation. This module provides the feature map of input image and the data compression using Discrete Wavelet Transform which is an optional step depending on the information quantity of this signature. The fully connected neural network, which has six hidden layers, classifies the image. With this technique, the computation time is reduced, and the network architecture is identical and simple independent of the type of dataset. The number of parameter is less than that in current research. The proposed method was validated with different dataset such as Caltech-101, Caltech-256, CIFAR-10, CIFAR-100, and ImageNet, and the accuracy reaches 92%, 90%, 99%, 94%, and 91%, respectively, which are better than the previous related works.

## 1. Introduction

For a software developer, it is a big challenge to search an image in database based on keyword, and the appropriate solution is to associate a label to all existing image. Finding a labelled image in database with table indexed facilitates the task. This operation of labeling is mainly called image classification which refers to a process in computer vision that can classify an image according To its visual content. Human visual is a perfect solution of image recognition; however, we cannot allocate a human resource to accomplish this task, and then automation is required.

The CNN or convolution neural network is categorized as a deep learning model, which is inspired by the organization of animal visual cortex used for processing data that has a grid pattern, such as images [1–3], and designed to automatically and adaptively learn spatial hierarchies of features from low- to high-level patterns. Convolution, pooling, and fully connected layers are the three types of layersthat constitute the CNN neural network. The feature extraction is ensured by convolution and pooling layers (first two layers), whereas the third, a fully connected layer, maps the extracted features into the final output for classification. The major recent works related to image classification use CNN to have a good result.

In 2014, GoogLeNet-19 developed by Google [4] was placed in first rank using 4 million parameters with a 6.67% of top-5 error rate, and in the second place, VGGNet-16, created by Simonyan, Zisserman [4] with 138 million parameters, and the top-5 error rate is 7.3%. It is evident that managing these parameters is difficult with a high number of layers. So, in this paper, we will propose an efficient approach with minimum computation time, minimum parameters, and minimum number of layers to classify images based on the light convolutional neural network (LCNN). To accomplish this, we suggest swapping the convolution and pooling layers of CNN with a single layer of pulse coupled neural network (PCNN) plus foveation contribution (when we visualize an image, we do not stare for longtime but we focus only on the pertinent information. It is a human cortex visual behavior called “foveation”) and an optional feature representation by the discrete wavelet transform (DWT). The fully connected layer remains the same but with minimum of neurons and hidden layers. To validate our method, we applied it to three databases with different classes and compare the result with several recent state-of-the-art methods. The main contributions of this work are cited as follows:

- The proposed image classification system has a simple architecture, and the topology remains unchanged, which is independent of image input, and due to this simplicity, the quantity of data to process is reduced compared with CNN, and it allows us to have an optimal computation time. Such kind of solution may be supported by embedded systems.
- Related to the first contribution, the approach works with minimum number of parameters, that is, less than 20.
- Foveation intervenes to collect the pertinent information to facilitate the construction of the image signature. It is a simple process compared with the succession of convolution and spooling operations used by CNN.
- DWT reduces the size (row × column) of image map in the aim to have a minimum number of neurons for the deep learning network.
- The approach provides high accuracy greater than or equal to the technique based on CNN, and even the proposed architecture is very simple.

The rest of the paper is organized as follows: Section 2 summarizes the recent works related to our proposed approach. The Section 3 describes the mathematic model of PCNN. The proposed method is the purpose of Section 4 followed by experimental results in Section 5 and discussion in Section 6. Finally, Section 7 concludes the paper with motivation. To ensure a good understanding of this paper, Table 1 presents the list of abbreviations and definitions.

## 2. Literature Review

Ferraz and Gonzaga [5] introduced a study focused on object classification based on local texture descriptor and a support vector machine. Recently, two new texture descriptors are proposed for object detection based on the Local Mapped Pattern (LPM) approach. The Center-Symmetric Local Mapped Pattern (CS-LMP) and Mean-Local Mapped Pattern (MLMP) exhibit better performance than SIFT and CS-LBP, but prior results have proven that the size of descriptors could be decreased without loss of sensitivity. In their research, they investigated the decreasing size of the M‐LMP descriptor, and the performance measurement was done by using the support vector machine (SVM) classifier for object classification. In those experiments, they applied an object recognition system based on the M-LMP reduced descriptor and compared those effects with the CS-LMP, Local Intensity Order Pattern (LIOP), and SIFT descriptors. The object classification outcomes analyzed the use of a Bag of Features (BoF) model and an SVM classifier, with the end result that overall performance using the reduced descriptor is higher than the other three well-known techniques tested and additionally requires less processing time. The experience was done with Caltech-101 and ImageNet dataset and the performance was good except with background Google class because the extraction feature drops some sensitive information and leads to the wrong deduction. This research can be compared with study done by Srivastava et al. [6] because both have the same objective and use a common Caltech-101 dataset to validate their experience. The last is a new concept of image classification using bag of LBP features constructed by clustering with fixed centers and SURF. This study presents a known approach for the variety of datasets having specific types of images. Hindi Signature, Bangla Signature, ORL Face, and Caltech-101 are the four datasets that are employed to validate the proposed classification method. The algorithm is spat into three steps as follows: the identification of Region of Interest (ROI) is the first step using SURF (Speed Up Robust Transform) Points, then LBP (Local Binary Pattern) extracts the features present in ROI as the second step, and the last step consists the clustering of LBP features which are done with a new proposed approach as CFC (Clustering with Fixed Centers) to construct Bag of LBP Features. Through proposed CFC technique, each image is tagged/annotated with a fixed Bag of Features to avoid the training of machine again and again. For image classification task, SVM intervenes because it has been experimentally found to give the best performance when compared with Random Forest, Decision Tree, Linear, K Nearest Neighbor, and Linear Method. The accuracy obtained for Signature (Bangla and Hindi), ORL, Face, and Caltech-101 is 87.0%, 81.6%, 75.0%, and 79.0%, respectively. Thus, the average accuracy obtained through the proposed approach is 81.7% in contrast to other state of art approaches having average accuracy as 64.15%, 76.47%, and 77.65%.

Han et al. [7] proposed a new CNN technique which could classify the images without difficulty compared to the other traditional models and gain better overall performance. With this method, the useful characteristic presentation of pretrained network can be efficaciously transferred to target task, and the original dataset can be augmented with the most treasured Internet images for classification. The method not only greatly reduces the requirement of a large training data but additionally effectively increases the training dataset. Both methods' capabilities make contributions to the considerable over-fitting reduction of deep CNNs on a small dataset. In addition, they successfully apply Bayesian optimization to remedy the tuff problem, hyper-parameter tuning, in network fine-tuning. The approach is applied to six public small datasets. Extensive experiments show that compared to conventional methods, the solution can help the famous deep-learning CNNs to achieve better performance. Specially, ResNet can outperform all the state-of-the-art models on six small datasets. The experiment results prove that the proposed solution can be a remarkable tool for dealing with practice problems that might be related to using deep CNNs on a small dataset; however, the accuracy decreases once the approach is applied to the large dataset or the dataset has many classes.

Çalik and Demirci [8] presented an image classification approach on embedded systems. The challenge was to apply CNN with device having a limited memory, and the result gives 85.9% accuracy using CIFAR-10 dataset with memory allocation of 2 GB. The limitation of this method is same as Srivastava et al. [6] research which has a difficulty to train through a big dataset. Dhouibi [9] published a paper-entitled optimization of the CNN model for image classification. It is talking about topology optimization of CNN in terms of number of layers and the number of neurons per layer. This optimal solution allows to reduce the model and enable to deploy it in embedded platforms. This research was experimented with the same previous dataset, and they obtained 82.43% accuracy. A third experience with the CIFAR-10 dataset is presented by Sharma and Phonsa [10]. They used the sequential method for the CNN and implemented the program in Jupiter notebook. They took 3 classes and classify them using CNN. The classes were airplane, bird, and car. They present the classification by using CNN, and they took batch size as 64. They got 94% accuracy for the 3 classes.

Wang and Sun [11] present a new method of image classification using CNN with wavelet domain inputs. The idea is to replace the first several convolutional layers part of feature extraction of standard CNN with wavelet packet transform or dual-tree complex wavelet transform. These wavelets transform allows to have a higher resolution of the image in preprocessing step. The advantage is to keep the essential information present in image to ensure a correct classification because with CNN, some important information may loss during convolution calculation. During the experience, Caltech-256 dataset and DTD dataset with ResNet-50 are used, and there is a maximum improvement of 2.15% and 10.26%, respectively, as accuracy.

Now, we are interested on the methods using ImageNet dataset qualified as largest image database on this area.

- Xception [12] or Extreme Inception is an improved version of the CNN inception model. Two levels are present on this conception as follows: the first level is composed by a single layer which slices the output into 3 segments and sent it to next filters. 1 *∗* 1, 3 *∗* 3 are, respectively, the convolution level of each filter. The depth-wise separable convolution [13–15] is the component which defines the Xception model. This technique intervenes in image classification with wide range of image having hundreds of classes (79% of accuracy for ImageNet dataset).
- VGG16 [12], which is inspired from AlexNet, has 16 layers and 3 fully connected layers. In the middle, there is 5 max pooling, and the Softmax is the output activation function [16–18] and ReLU for hidden layers. VGG19 [19] has a same concept as VGG16; however, this CNN contains 19 layers with 3 fully connected layers for classification and 16 convolution layers for feature extraction. The accuracy top-1 score for both is 71.3%.
- ResNet152V2 and MobileNetV2 [20] are well-known as CNNs for pretrained deep learning. They are specialized on feature extraction, prediction, and classification. A fully convolution layer through 32 filters and 19 residual bottleneck layers forms the architecture model of MobileNetV2. Concerning the ResNet152V2, it has thousands or hundreds of convolution layers, and the particularity compared with the previous version is that it employs a normalization batch before each weight layer. 78.0% and 71.3% are the recognition rate got with ImageNet dataset.
- NASNetLarge is a generation of CNN having a capacity to train more than a million pictures from ImageNet dataset and classify more than thousand objects. An input image of this network has 331 × 311 size and the strong point of this concept is that it has learned rich feature representations for a wide range of images. The experience is showing that the final accuracy rate reaches 82.5%. On the other hand, 84.3% is the performance using EfficientNetB7 [21]. EfficientNetB7 is a release of EfficientNet which is a lightweight NAS-based network created by Google in 2019.

The common point of these studies is the ambition to optimize the standard CNN. Each research has its own methodology to extract image feature to reach the goal. Concerning the classification layer, some stay with one or more fully connected neural networks and the other tries to intervene SVM. They are selected as part of state of the art in this paper because the objective is similar even the experimental dataset then we have a possibility to compare the performance.

## 3. Pulse Coupled Neural Network

According to Srinivasan et al. [22] presentation, PCNN is inspired from behaviors of cat visual cortex phenomena. The modelling architecture is composed of three parts, namely, the dendritic tree, the linking modulation, and the pulse generator. The first part has two types of entries, namely, feeding and linking. The feeding receives the local and external stimulus; however, the linking captures the local only. The second part, which is the linking modulation, combines the outputs from two channels by adding a bias to the linking and multiplying it with feeding. Internal state of neuron *U*_*j*_ is the result of such combination, and this internal state and the threshold help the last part pulse generator to generate the pulse.

Lo et al. [23] introduce PCNN in image processing area and the mathematics modelling is defined below. The Table 2 explains the meaning of different parameters in PCNN.

(i) First part (dendritic tree):
  (1) $$\begin{array}{c} F_{ij}\left[n\right]=\exp\;\left(-\alpha_{F}\delta_{n}\right)F_{ij}\left[n-1\right]+S_{ij}+V_{F}\underset{kl}{\sum }M_{ijkl}Y_{kl}\left[n-1\right]\text{,}\\ L_{ij}\left[n\right]=\exp\;\left(-\alpha_{L}\delta_{n}\right)L_{ij}\left[n-1\right]+V_{L}\underset{kl}{\sum }W_{ijkl}Y_{kl}\left[n-1\right]\text{.} \end{array}$$
(ii) Second part (linking modulation):
  (2) $$\begin{array}{c} U_{ij}\left[n\right]=F_{ij}\left[n\right]\left(1+\beta .L_{ij}\left[n\right]\right)\text{.} \end{array}$$
(iii) Last part (pulse generator):
The internal state of the neuron is compared to a dynamic threshold, Θ, to produce the output, *Y*, by
  (3) $$\begin{array}{c} Y_{ij}\left[n\right]=\left\{\begin{array}{cc} 1\text{,} & if\;U_{ij}\left[n\right]>\Theta_{ij}\left[n\right]\text{,}\\ 0\text{,} & Otherwise\text{.} \end{array}\right. \end{array}$$

The threshold is dynamic in that when the neuron fires (*Y* > Θ) the threshold then significantly increases its value [23]. This value then decays until the neuron fires again. This process is described by

(4) $$\begin{array}{c} \Theta_{ij}\left[n\right]=\Theta_{ij}\left[n-1\right]\exp\;\left({-\alpha }_{\Theta }\right)+V_{\Theta }Y_{ij}\left[n-1\right]\text{.} \end{array}$$

According to equation (3), the output is binary and then there is a lot of candidates for the foveation points because with standard PCNN, a threshold function having output 0 or 1 is used by the pulse generator module. This issue can be solved by adapting the sigmoid pulse generator as defined in equation (5) [24, 25] as given as follows:

(5) $$\begin{array}{c} Y_{ij}\left[n\right]=\frac{1}{1+\exp\;\left[-\Upsilon \left(U_{ij}\left[n\right]-\Theta_{ij}\left[n-1\right]\right)\right]}\text{.} \end{array}$$

**Figure 1:**
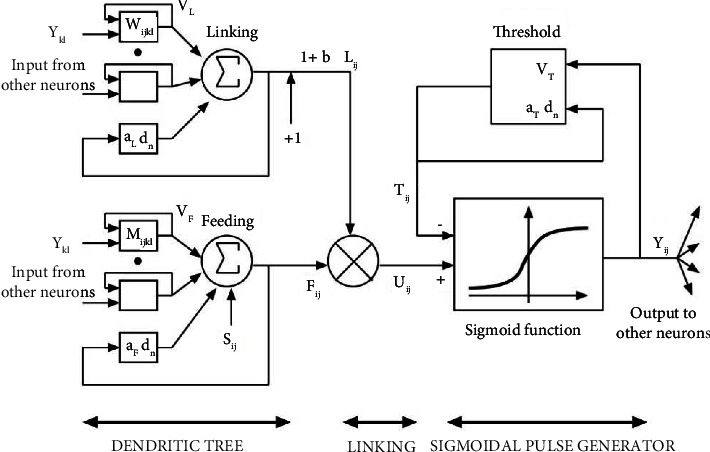
Outline of PCNN with the sigmoidal pulse generator.

Figure 1 represents the described model and the output varies from 0 to 1 [26].

## 4. Proposed Method

**Figure 2:**
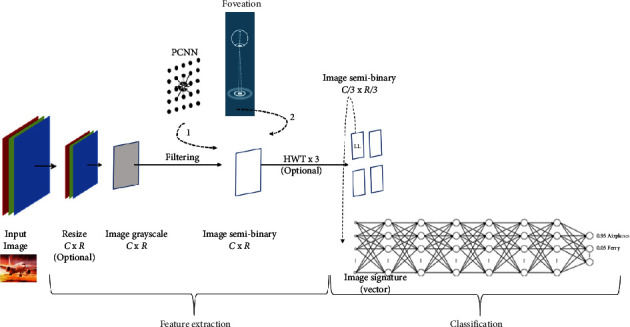
Architecture of proposed method.

Now, we have more visibility about PCNN which is an element involved in the image classification method. The wavelet transforms and fully connected neural network (FCNN) will be explained briefly during these interventions in the approach. The proposed system has two modules, namely, feature extraction and deep learning module, and a clear presentation of the approach is shown in Figure 2.

### 4.1. Feature Extraction

**Figure 3:**
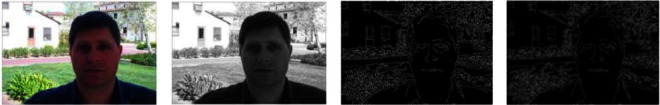
(a) Original image- (b) grayscale image- (c) canny filter result- (d) blurring filter.

First step is to choose the image dataset and split it in two parts, namely, training and validation. All existing image in database must be converted to grayscale and resized (optional) because PCNN can process only a matrix with one dimension instead of three like an RGB image. Image resizing is applicable only when the image has a large dimension. A part of color conversion, preprocessing module, has two filters, namely, Canny and blurring filter. The reason of this choice is to reduce the quantity of information to be processed. Canny filter is an edge detection operator that uses a multistage algorithm to detect a wide range of edges in images. It was developed by John F. Canny [27] in 1986. Blurring filter [27, 28] is a low pass filter, because it allows low frequency to enter and stop high frequency. Here, frequency means the change of pixel value. Around edge pixel, value changes rapidly as blur image is smooth; so high frequency should be filtered out. The Figure 3 represents such details.

PCNN extracts the essential part from blurring image and eliminates the noise background. High number of iterations is required to ensure that PCNN accomplishes his task. Before starting the iteration, we should initiate the neural network parameters as follows:

(i) Weights matrix
  (6) $$\begin{array}{c} M=W=\left[\begin{array}{ccc} 0.707 & 1 & 0.707\\ 1 & 1 & 1\\ 0.707 & 1 & 0.707 \end{array}\right]\text{.} \end{array}$$
(ii) Initial values of matrix
The preliminary values of linking *L*, feeding *F* matrix, and stimulus *S* are similar to the enter image. The convolution among null matrix which has the same length as the enter image *R *×* C* and weights matrix initiates the output value *Y* of PCNN. The initial value of dynamic threshold Θ is an *R*-by-*C* matrix of two.
(iii) Constants delay
  (7) $$\begin{array}{c} \alpha_{F}=0.1,\alpha_{\theta }=1,\alpha_{L}=1.2\text{.} \end{array}$$
(iv) Constants normalization
  (8) $$\begin{array}{c} V_{F}=0.5{,V}_{\Theta }=20,V_{L}=0.2,\Upsilon =0.9,\beta =0.1\text{.} \end{array}$$

**Figure 4:**
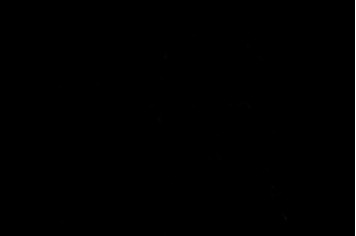
PCNN image output.

The maximum number of iterations is fixed to 40 and the calculation of the percentage of misclassified pixel [29] indicates the image to be selected. The first minimum rate corresponds to excellent image segmentation and the second to edge detection, so we are interested in the second result shown in Figure 4. Its gray level varies between 0 and 1 due to the sigmoidal pulse generator used by the PCNN neural network.

**Figure 5:**
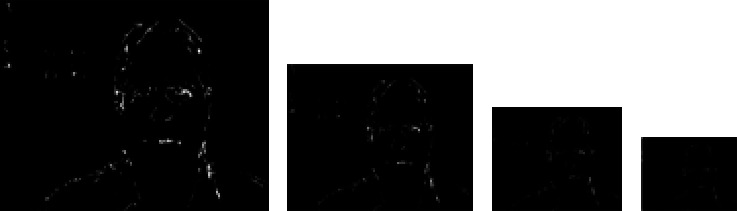
(a) Foveation image- (b) LL of level 1 HWT- (c) LL of level 2 HWT- (d) LL of level 3 HWT.

PCNN task is completed by extracting the relevant information. Currently, we solicit the foveation method to collect the data sensitive to human eyes. For this, we apply an image threshold and we have the result shown in Figure 5(a).

**Figure 6:**
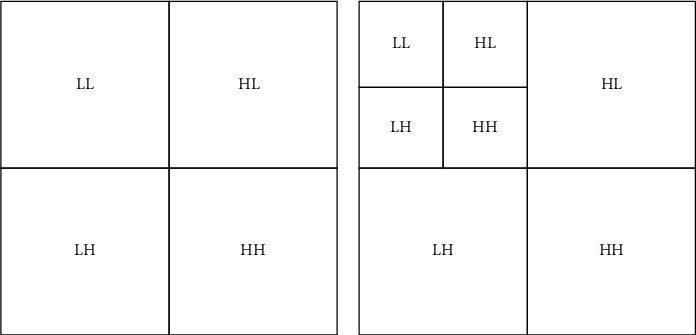
Structure of Haar wavelet decomposition. (a) Decomposition level 1. (b) Decomposition level 2.

Now, we should reduce the dimension of the image (this step is optional if the image has a small size like 32 × 32), and it can be done by Haar Wavelet Transform (HWT). HWT operates simultaneously in spatial and frequency domain information in image processing. It is a transform for which the wavelets are sampled at discrete intervals [30, 31]. Haar wavelet operates on data by calculating the sums and differences of adjacent elements. To apply HWT on images, a simple explanation is shown in Figure 6. Four subbands, namely, *LL*, *HL*, *LH*, and *HH* subbands (*L* = Low, *H* = High) compose the resulting image where *LL*-subband contains an approximation of the authentic image while the other subbands comprise the missing details. The *LL*-subband output from any stage can be decomposed similarly [32].

We apply HWT transform three times to the foveation image, and we are interested on the second LL-subband (in Figures 5(b)–5(d)). The resulting image will be reshaped to vector to constitute the value of input layer of FCNN.

### 4.2. Classification

FCNN has three parts, namely, input, hidden, and output layers. As the name is called fully connected, it means that each neuron connects to all neurons existing in the next layer. Before going to the activation function, the computation of input, weight, and bias must be done beforehand. We focus only on two activation functions, namely, the nonlinear ReLU function and softmax function. They are defined in equations (9) and (10).

(9) $$\begin{array}{c} f_{ReLU}\left(x_{i}\right)=\left\{\begin{array}{cc} 0\text{,} & if\;x_{i}<0\text{,}\\ x_{i}\text{,} & if\;x_{i}\geq 0\text{,} \end{array}\right. \end{array}$$

(10) $$\begin{array}{c} f_{softmax}\left(x_{i}\right)=\frac{e^{x_{i}}}{\overset{N}{\underset{j=1}{\sum }}e^{x_{j}}}\text{,} \end{array}$$

where *x*_*i*_ is the sum inputs improved by means of weights plus bias and *N* the number of neurons in the output layer. The value of the ReLU function is 0 or *x*_*i*_, and for softmax function, it is between 0 and 1 because it is indicating the probability that in which class the image belongs. The feature map of the input image constitutes the input layer (size of image signature × 1), and the image class membership forms the output layer [33].

Six hidden layers are required at least and, in this paper, we fix it to 6. The activation characteristic for them is the ReLU function, and all weights are initialized randomly. It means that there are six weights, namely, *w*_1_(*h*_1_, 1024), *w*_2_(*h*_2_, *h*_1_), *w*_3_(*h*_3_, *h*_2_), *w*_4_(*h*_4_, *h*_3_), *w*_5_(*h*_5_, *h*_4_), *w*_6_(*class* *number*, *h*_5_) where (*h*_*j*_ × *h*_*k*_) is the size of weight *w*_*i*_. For experience purpose, the value of *h* is a square root of size of image signature and number of classes. Concerning output layer (n*umber* *of* *classes* *x* 1), the number of neurons is the same as the number of classes present in dataset. The neuron which has a high probability value determinates the belonging class. The activation function softmax ensures this probability format. Evidently, the number of neurons in input layer is equivalent to the length of image signature vector. The percentage of image allocated for testing depends on the searcher choice but it is important to have a percentage training dataset more than testing images. During training phase, the output neuron corresponding to input image signature is 1 and 0 for leftovers.

## 5. Experiments

To evaluate the performance of the proposed method, we introduce three datasets that are used by different research cited in literature review Section 2 in the aim to compare these performances with ours. They are publicly available. The Section 4.1 describes the content of each dataset and Section 4.2 details the performance using image classification measurement like accuracy [34], loss [35], precision, recall, and F1 score [36, 37].

### 5.1. Dataset Description

**Figure 7:**
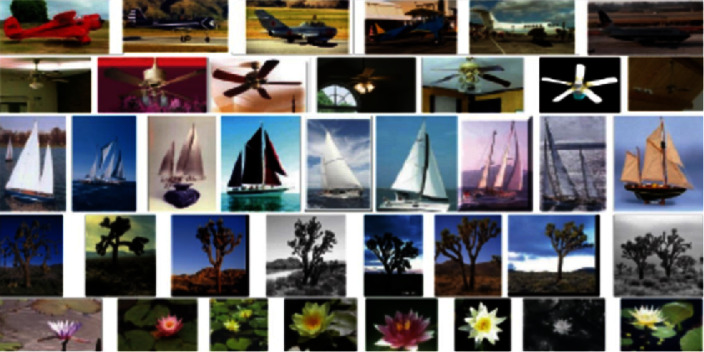
Sample images from caltech-101 dataset.

Caltech-101 (The dataset is available at https://www.kaggle.com/datasets/862ae86edba271c39f76d0b530edeb55076b4b82b971160637210900747c44b1) is the first image dataset that we use to test our conception. It includes photos of gadgets belonging to 101 classes plus one background clutter class. Every photo is labelled with single item and every class carries kind of forty to 800 pics, totaling to 9146 photos. We are not able to show here all content of this dataset; however, a sample of images is presented in Figure 7 [24].

**Figure 8:**
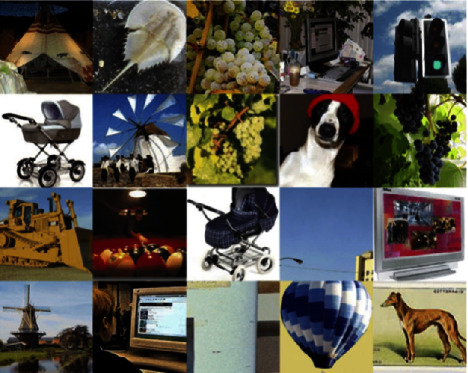
Sample images from caltech-256 dataset.

The second dataset is Caltech-256 (The dataset is available at https://www.kaggle.com/datasets/jessicali9530/caltech256) dataset [38] having 30607 natural photographs, consisting of 256 object categories and 1 random background class. The common variety of photos in every class is 119 (variety from eighty to 827) and the average photo dimension is 371 × 326. A sample snapshot is presented in Figure 8.

**Figure 9:**
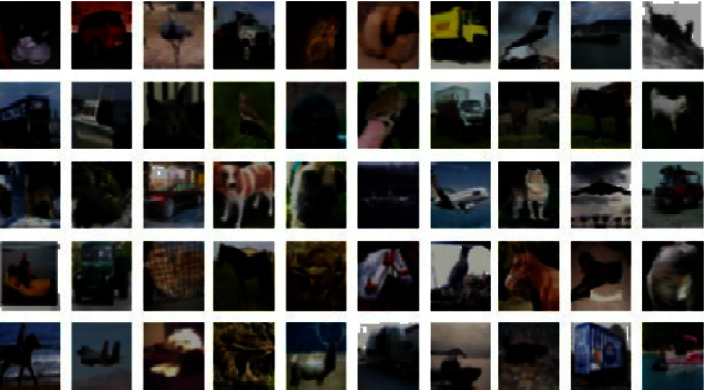
Sample images from CIFAR-10 dataset.

The third dataset that we use for testing is CIFAR-10 (The dataset is available at https://www.cs.toronto.edu/~kriz/cifar.html) (Canadian Institute for Advanced Research, 10 classes). This dataset contains 60000 32 × 32 color images divided into 10 classes (airplane, automobile, bird, cat, deer, dog, frog, horse, ship, and truck), each with 6000 images [39].  Figure 9 shows a few sample images from the CIFAR-10 dataset.

**Figure 10:**
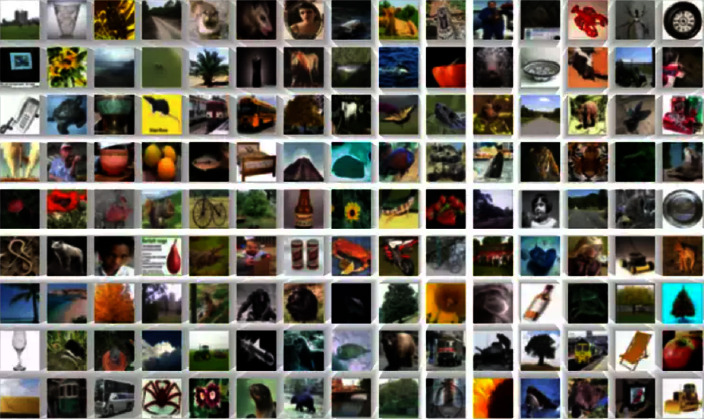
Sample images from CIFAR-100 dataset.

The fourth dataset is CIFAR-100 (The dataset is available at https://www.cs.toronto.edu/~kriz/cifar.html) which is similar to the CIFAR-10, except it has 600 images for each class (100 classes in total). In CIFAR-100, there are 20 super classes subgrouped into 100 classes. The dataset comes with two labels for each image such as a “fine” label (class) and a “coarse” label (superclass). A sample of images present in this dataset is shown in Figure 10.

**Figure 11:**
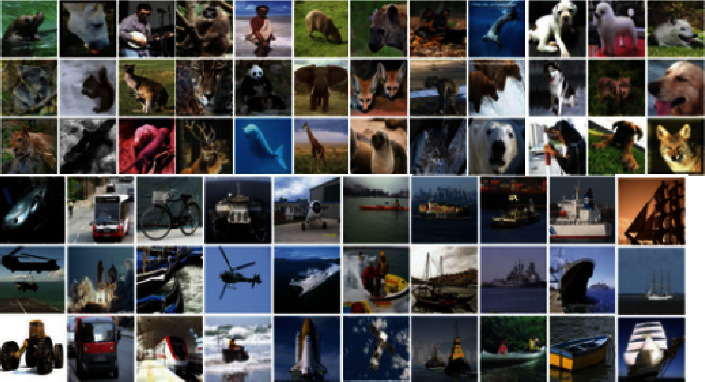
Sample images from imageNet dataset.

The last dataset for experiments is ImageNet (The dataset is available at https://www.image-net.org/download.php). It is a wide database having more than one million images and spans 1000 object classes. ImageNet dataset is publicly available and a snap shot is shown in Figure 11.

### 5.2. Performance Measurement

**Figure 12:**
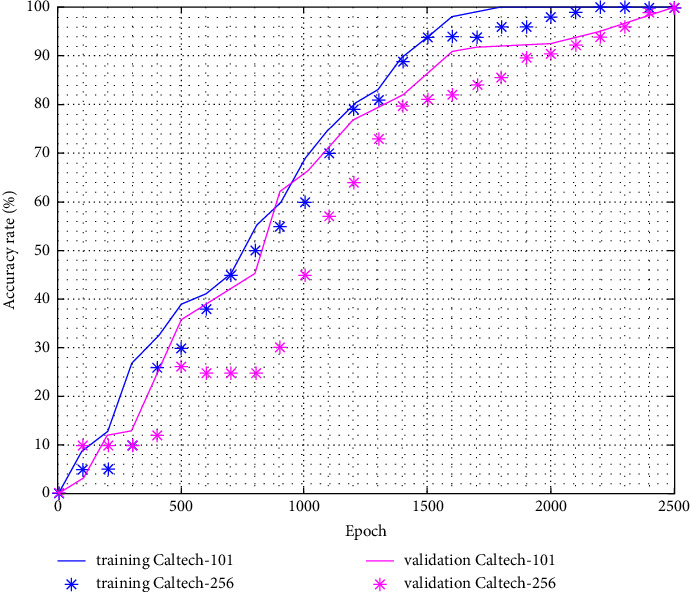
Top-1 accuracy for caltech-101/256 datasets.

We fix the number of epochs to 2500, it does not depend on dataset but it can be increased to improve the accuracy. The first experience was done with Caltech-101 dataset that 75% of image will be processed for training purpose and 25% (2279 images) of remaining dataset will pass through our network for validation. It means that we test 25% for each class. The dataset split must be the same as used by previous studies; otherwise, we cannot compare the result. The accuracy average is around 91%, and the sample of performance is the object of Table 3. The precision is excellent when the number of images belonging to a class is not high. We remark also that the accuracy commences acceptable when reaching 1500th epoch according to the Figure 12. Concerning the loss, it converges to null once the epoch is near to 1700.

**Figure 13:**
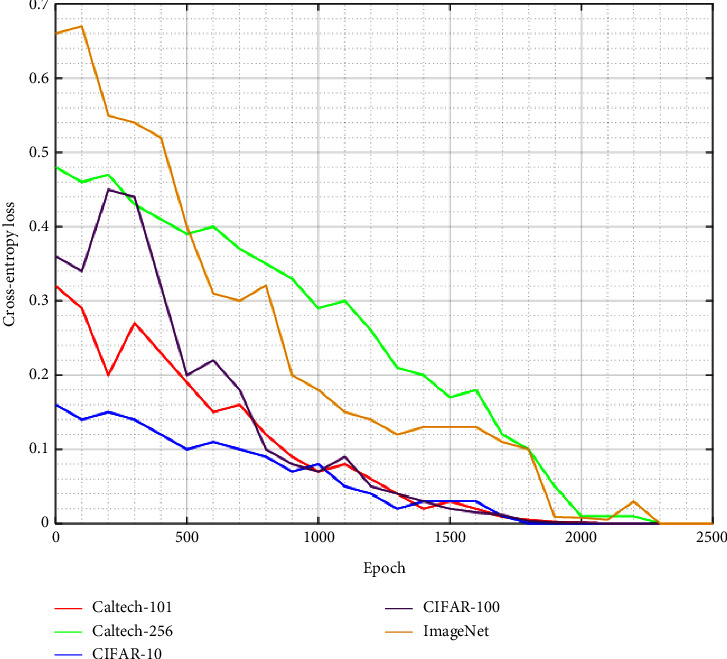
Loss experience value for five datasets.

The Caltech-256 is considered an improvement to its predecessor, the Caltech 101 dataset, with new features such as larger category sizes, new and larger clutter categories, and overall increased difficulty. The accuracy is reduced 2% compared with Caltech-101 (Figure 12) because the number of class is increased; however, the performance is better if the number of images in one class is large. We can observe it for motorbikes experience (Table 4). The loss value is considerable until the end of experience (Figure 13). To fix this issue, it is possible to augment the number of epochs but it will have an impact on the other parameter. For precision, the loss function used is the cross entropy as defined in (11)

(11) $$\begin{array}{c} L_{CE}=-\overset{N}{\underset{i=1}{\sum }}t_{i}\log\left(p_{i}\right)\text{,} \end{array}$$

where *t*_*i*_ is the truth label, *p*_*i*_ the softmax probability for the *i*^*th*^ class and *N*, the number of image class present in dataset [40].

**Figure 14:**
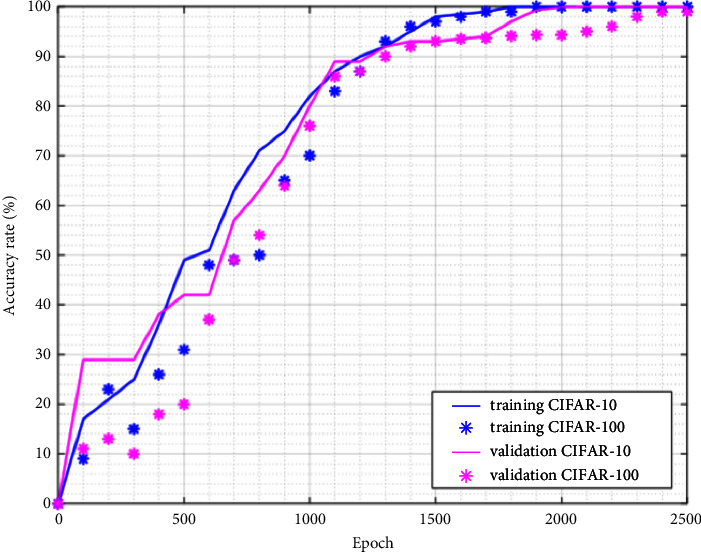
Top-1 accuracy for CIFAR-10/100 datasets.

The experience with CIFAR-10 is rapid because the number of classes is less which is why the accuracy rate is high from 1000^th^ epoch. Resizing image and HWT is not required because the image has a small dimension (32 × 32). We select 50000 images (90%) for training and 10000 images (10%) for testing. This partition is the common partition used by previous researchers' works. Same as proceed with Caltech-256, the full result is presented in Table 5 which provides the accuracy details for each class. Regarding the epoch, it is shown in Figure 14. The proposed method by Sharma and Phonsa [10] was tested with 3 classes, namely, aeroplane, bird, and cat. If we limit only our test with these classes, we got an accuracy of 99%.

**Figure 15:**
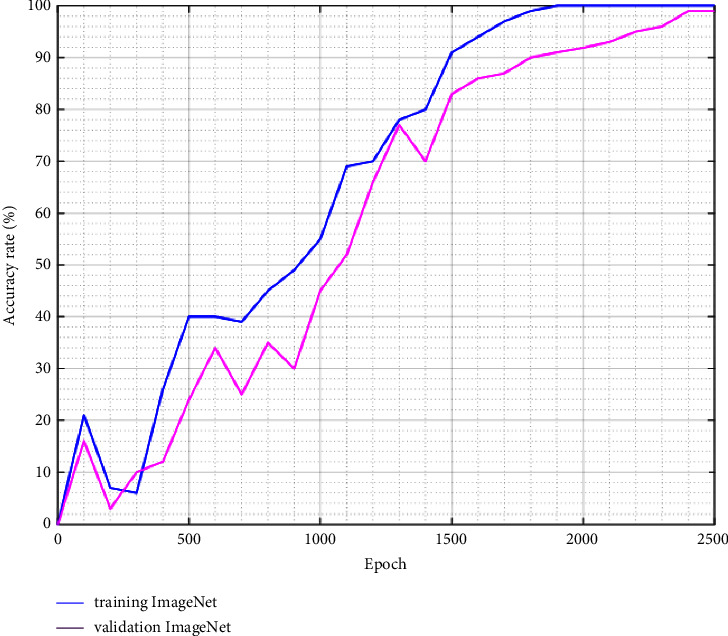
Top-1 accuracy for imageNet dataset.

Now, we test the technique with largest image dataset like CIFAR-100 and ImageNet. The performance is reduced because the dataset has many classes and the number of images for testing is less too (Figures 14 and 15). It can be improved by increasing the number of epochs; however, it may have an impact in computational time. To support such suggestion with the embedded system, a device having a good configuration is necessary. As we see in Figure 13, the loss function starts with highest value and it becomes negligible at the end of the epoch. The cross-entropy trend for both datasets is different comparing with three previous ones. The experience metrics are presented in Tables 6 and 7, and we notice that our accuracy is still competitive.

Most of image classification research studies based on CNN use ImageNet as dataset, and we will compare these performances with ours using the same device configuration which is described as follows:

- CPU: AMD EPYC Processor (with IBPB) (92 core)
- RAM: 1.7T
- GPU: Tesla A100
- Batch size: 32

As a part of top-1 accuracy, we compare also the top-5 accuracy, number of parameters, and computation time per each method in Table 8. We remark that our proposed method has a good performance. With another device having a limited memory like embedded systems 2 GB, the computation time augments but is still tolerable. The research done by Çalik and Demirci [8] is dedicated for small dataset (CIFAR-10); however, we have high rate of recognition 85.9% vs 99.11%.

Before closing this paragraph, we confront our result with some research studies using a smallest dataset such as Caltech-101, Caltech-256, CIFAR-10, and CIFAR-100 (Table 9). We see that the proposed approach leads the performance except for Caltech-256 experience in which we are on the second position. The symbol «−» in tables means that that the authors did not provide the information in these paper publications and «*∗*», the maximum value.

## 6. Discussion

Most of recent research in image classification choose CNN as a neural network to accomplish the task. It collects the relevant information in feature layer which is the estate of convolution and pooling. Both operations reduce the volume of information to be processed and the final important information jugged essential called image map or signature is going through fully connected neural network for image classification purpose. This technique required from thousand to million parameters, and the architecture changes according to the dataset to be treated. It means that the solution is complex and may have an impact on the performance. For this reason, we propose this approach with 11000 parameters maximum and simple/static architecture, and the accuracy is improved. Such result was due to the foveation produced by PCNN. The methods that are CNN-based have a facility to classify an image containing a background because they give an importance on such information; however, ours has a weakness which is why the accuracy for the background class in Caltech-101 dataset is less (85%) because the PCNN ignores this information. Here, we are talking about top-1 accuracy but the top-5 accuracy is at 90%.

Regarding the test with CIFAR-10 image dataset, the approach proposed by Sharma and Phonsa [10] has an accuracy less than ours, and even the number of classes is less because the dataset has only ten classes and the image inside does not have a large dimension. Different type of image like dog and cat may not have similar signature due to foveation which is why the performance is always high. So, we advise people to choose our approach if the number of image's class is less, and with no much background, even image dimension is considerable. Otherwise, a large number of epochs is recommended for a large dataset like Caltech-256, CIFAR-100, and ImageNet. MCNN and CNN for image classification are complementary to ensure an excellent result. A module of preprocessing should be added in chain of processing to decide in which case the system uses convolution/pooling or PCNN/foveation as the feature extraction layer.

Before concluding this paper, we resume in Table 10 the advantage and disadvantage for each algorithm.

## 7. Research Motivation and Conclusion

Applying CNN for image classification demands high number of parameters and the feature extraction layers require a big computing resource for getting an image map, and this step may cause a delay in processing. So, the first motivation of this research is to propose a simple architecture and a simple static model independent of input image or dataset with minimum computation time. The second motivation is to have a neural network more efficient with an accuracy more than existing image recognition algorithms. To attend on these objectives, we resize if the image has a large dimension and converts to gray level before PCNN and foveation processing. The resulting image goes through wavelet transformation in the three level by keeping the final approximation matrix for the FCNN input layer. This transformation reduced the information with minimum loss. For validation, we choose five datasets, namely, Caltech-101, Caltech-256, CIFAR-10, CIFAR-100, and ImageNet and comparing the existing methods with same dataset, the proposed method has a good performance especially with small dataset like CIFAR-10.

PCNN always keeps an unmissable step in image processing area, and foveation is an application of this intelligent neural network. Aside searching picture in database, we are able to apply this approach in face recognition and finger print recognition, for example. The axe improvement of this study may be oriented to replace the PCNN model with modified pulse coupled neural networks (MPCNN) or intersecting cortical version (ICM). Three works [42–44] are published recently, and they can be a source of inspiration to improve this research. In future work, we focus only in two class of images, namely, person with and without facemask, and in case of image with facemask, we will proceed to check whether the mask is worn correctly.

## Figures and Tables

**Table 1:** Table of abbreviations.

Abbreviation	Definition
BoF	Bag of features
CFC	Clustering with fixed centers
CNN	Convolutional neural network
CS-LBP	Center symmetric local binary pattern
CS-LMP	Center symmetric local mapped pattern
DTD	Describable textures dataset
DWT	Discrete wavelet transform
FCNN	Fully connected neural network
HWT	Haar wavelet transform
ICM	Intersecting cortical model
LBP	Local binary pattern
LCNN	Light convolutional neural network
LIOP	Local intensity order pattern
LMP	Local mapped pattern
MLMP	Mean-local mapped pattern
MPCNN	Modified pulse coupled neural networks
NASNet	Neural architecture search network
PCNN	Pulse coupled neural network
ReLU	Rectified linear unit
ResNet	Residual network
ROI	Region of interest
SIFT	Scale invariant feature transform
SURF	Speed up robust transform
SVM	Support vector machine
VGG	Visual geometry group

**Table 2:** PCNN equations parameter.

Where	Is
Θ_*ij*_	Dynamic threshold
*F*_*ij*_	The feeding compartment of the (*i*, *j*) neuron embedded in a 2D array of neurons
*L*_*ij*_	The corresponding linking compartment
*S*_*ij*_	Input stimulus
*U*_*ij*_	Internal activity of neurons
*V*_Θ_	Large constant
*Y*_*kl*_	Neuron's outputs from a previous iteration (*n* − 1)
*α*_*F*_, *α*_*θ*_, *α*_*L*_	Time constant where *α*_*F*_ < *α*_*θ*_ < *α*_*L*_
*M*, *W*	Constant synaptic weights
Υ	Sigmoid function
*β*	Linking strength

**Table 3:** Sample performance metrics for caltech-101.

Class	Number of images	Precision	Recall	F1	Accuracy (top-1)
Airplanes	800	0.9821	0.9821	0.9821	0.9651
Bonsai	128	0.9750	0.9750	0.9750	0.9535
Camera	50	1.0000	0.9375	0.9677	0.9444
Cannon	43	1.0000	0.9286	0.9630	0.9375
Dalmatian	67	0.9500	1.0000	0.9744	0.9545
Elephant	64	0.9474	0.9474	0.9474	0.9091
Euphonium	64	1.0000	0.9500	0.9744	0.9545
Faces	435	0.9923	0.9923	0.9923	0.9850
Pizza	53	1.0000	0.9412	0.9697	0.9474
Soccer_ball	64	1.0000	0.9500	0.9744	0.9545
Sunflower	85	0.9615	0.9615	0.9615	0.9310
Total	9115	0.9722	0.9437	0.9571	0.9270

**Table 4:** Sample performance metrics for caltech-256.

Class	Number of images	Precision	Recall	F1	Accuracy (top-1)
002. American-flag	78	0.8750	0.9545	0.9130	0.8519
046. Computer-monitor	106	0.9688	0.8857	0.9254	0.8684
064. Elephant-101	105	0.9688	0.9688	0.9688	0.9429
105. Horse	216	0.9077	0.9833	0.9440	0.8971
127. Laptop-101	102	0.9355	0.8788	0.9063	0.8286
145. Motorbikes-101	638	0.9948	0.9694	0.9819	0.9645
232. T-shirt	286	0.9535	0.8913	0.9213	0.8571
240. Watch-101	161	0.9167	0.9778	0.9462	0.9020
251. Airplanes-101	640	0.9792	0.9947	0.9869	0.9744
253. Faces-easy-101	348	0.9519	0.9900	0.9706	0.9439
Total	2680	0.9452	0.9494	0.9464	0.9031

**Table 5:** Performance metrics for CIFAR-10.

Class	Number of images	Precision	Recall	F1	Accuracy (top-1)
Airplane	6000	0.9859	1.0000	0.9929	0.9859
Automobile	6000	0.9946	0.9957	0.9952	0.9904
Bird	6000	0.9958	0.9990	0.9974	0.9948
Cat	6000	0.9969	0.9990	0.9979	0.9959
Deer	6000	0.9947	0.9895	0.9921	0.9844
Dog	6000	0.9959	0.9990	0.9975	0.9949
Frog	6000	0.9895	0.9989	0.9942	0.9885
Horse	6000	0.9950	0.9990	0.9970	0.9940
Ship	6000	0.9894	0.9989	0.9941	0.9883
Truck	6000	0.9949	0.9990	0.9969	0.9939
Total	60000	0.9933	0.9978	0.9955	0.9911

**Table 6:** Sample performance metrics for CIFAR-100.

Class	Number of images	Precision	Recall	F1	Accuracy (top-1)
Beaver	600	0.9479	1.0000	0.9733	0.9490
Aquarium fish	600	0.9892	0.9583	0.9735	0.9495
Orchids	600	0.9677	0.9890	0.9783	0.9583
Bottles	600	0.9583	0.9892	0.9735	0.9495
Apples	600	0.9381	0.9891	0.9630	0.9300
Clock	600	0.9596	0.9896	0.9744	0.9510
Bed	600	0.9551	0.9884	0.9714	0.9457
Bee	600	0.9293	0.9892	0.9583	0.9216
Baby	600	0.9574	0.9890	0.9730	0.9485
Crocodile	600	0.9444	0.9884	0.9659	0.9355
Total	6000	0.9547	0.9870	0.9705	0.9438

**Table 7:** Sample performance metrics for imageNet.

Class Id	Class name	Precision	Recall	F1	Accuracy (top-1)
36	n016677—8-terrapin	0.9302	0.9756	0.9524	0.9130
113	n019443—0-snail	0.9756	0.9091	0.9412	0.8936
157	n020869—0-papillon	0.9318	0.9762	0.9535	0.9149
292	n021296—4-tiger	0.9219	0.9833	0.9516	0.9104
430	n028024—6-basketball	0.9643	0.9310	0.9474	0.9000
448	n028436—4-birdhouse	0.9500	0.9694	0.9596	0.9223
478	n029713—6-carton	0.9770	0.9444	0.9605	0.9247
650	n037599—4-microphone	0.9216	0.9895	0.9543	0.9143
677	n038047—4-nail	0.9565	0.9888	0.9724	0.9474
889	n045368—6-violin	0.9674	0.9889	0.9780	0.9579
Total		0.9496	0.9656	0.9571	0.9199

**Table 8:** ImageNet performance metrics comparison.

Model	Accuracy (top-1)	Accuracy (top-2)	Parameters	Time (ms) per inference step (CPU)	Time (ms) per inference step (GPU)
Xception [12]	0.7900	0.9450	22.9 M	109.4	8.1
VGG16 [12]	0.7130	0.9010	138.4 M	69.5	4.2
VGG19 [19]	0.7130	0.9000	143.7 M	84.8	4.4
ResNet152V2 [20]	0.7800	0.9420	60.4 M	107.5	6.6
MobileNetV2 [20]	0.7130	0.9010	3.5 M	25.9	3.8
NASNetLarge [21]	0.8250	0.9600	88.9 M	344.5	20.0
EfficientNetB7 [21]	0.8430	0.9700	66.7 M	1578.9	61.6
Proposed method	0.9199	0.9573	201019	25.1	3.0

**Table 9:** Performance comparison.

Technique	Year	Number of parameters	Dataset	Precision	Recall	F1	Accuracy (top-1)
Local texture descriptor + SVM [5]	2017	—	Caltech-101	—	—	—	0.7770
CNN applicable in small dataset [8]	2018	—	CIFAR-10	—	—	—	0.8590
Bag of LBP + SVM [6]	2019	—	Caltech-101	0.6300	0.6100	0.6100	0.7900
Standard CNN [38]	2019	—	Caltech-256	0.95	0.96	0.9549	0.9600
Optimization CNN model [9]	2021	2915114	CIFAR-10	—	—	—	0.8240
CNN sequential method [10]	2021	289443	CIFAR-10	—	High	—	0.9420
ResNet50 [41]	2021	—	Caltech-101/Caltech-256/CIFAR-10	—	—	—	0.6852/0.8040/0.9079/
CNN + DWT [11]	2022	—	Caltech-256	—	—	—	0.7224
Proposed method	2022	10980^*∗*^	Caltech-101/Caltech-256/CIFAR-10/CIFAR-100	0.9722/0.9452/0.9933/0.9547	0.9437/0.9494/0.9978/0.9870	0.9571/0.9464/0.9955/0.9705	0.9270/0.9031/0.9911/0.9438

**Table 10:** Advantage and disadvantage of each methods.

Year	Paper	Advantage	Disadvantage
2017	[5]	(i) High speed of processing	(i) A lot of parameters required for training
		(ii) High accuracy	
		(iii) Having an ability to intervene in big dataset images	(ii) Network architecture complex

2018	[8]	(i) Less processing times	The algorithm is dedicated for a small dataset like CIFAR-10; otherwise, the performance is not considerable
		(ii) High accuracy with small image	

2019	[6]	Highest accuracy for face image category	(i) Long chain of processing before classification
			(ii) Lowest accuracy for classifying an image with a variant content like pizza category

2021	[9]	(i) Minimum number of epochs	(i) A million of parameters
		(ii) High accuracy with image having small size	(ii) Weakness with dataset having large image

2021	[10]	Minimum time of training	Low accuracy for a dataset with many classes

2021	[41]	High accuracy	(i) Maximum number of parameters and epochs
			(ii) High computation time

2022	[11]	(i) Maximum quantity of information in image signature	(i) Training time around 13 hours
		(ii) Medium accuracy rate (top-1)	(ii) Accuracy improved observed only for top-5 accuracy measurement

—	[12, 19–21]	(i) Good accuracy	(i) Too much parameters
		(ii) Minimum computation time	

2022	Proposed method	(i) Minimum parameters required	(i) Number of epochs maximum is required
		(ii) Minimum computation time (2.11 milliseconds for an image with small size (32 × 32))	
		(iii) The architecture is always the same independently of image dataset	(ii) A bit difficulty to classify an image having important background

## Data Availability

The data used to support the findings of this study are available from the corresponding author upon request.

## References

[1] Yamashita R., Nishio M., Do R. K. G., Togashi K. (2018). Convolutional neural networks: an overview and application in radiology. *Insights Imaging*.

[2] Hubel D. H., Wiesel T. N. (1968). Receptive fields and functional architecture of monkey striate cortex. *Journal of Physiology*.

[3] Fukushima K. (1980). Neocognitron: a self-organizing neural network model for a mechanism of pattern recognition unaffected by shift in position. *Biological Cybernetics*.

[4] Analytics Vidhya (2020). The Development of Visual Priors Across the Lifespan. https://medium.com/analytics-vidhya/cnns-architectures-lenet-alexnet-vgg-googlenet-resnet-and-more-666091488df5.

[5] Ferraz C. T., Gonzaga A. (2017). Object classification using a local texture descriptor and a support vector machine. *Multimedia Tools and Applications*.

[6] Srivastava D., Bakthula R., Agarwal S. (2019). Image classification using SURF and bag of LBP features constructed by clustering with fixed centers. *Multimedia Tools and Applications*.

[7] Han D., Liu Q., Fan W. (2018). A new image classification method using CNN transfer learning and web data augmentation. *Expert Systems with Applications*.

[8] Çalik R. C., Demirci M. F. Cifar-10 image classification with convolutional neural networks for embedded systems.

[9] Dhouibi M. Optimization of CNN model for image classification.

[10] Sharma A., Phonsa G. Image classification using CNN.

[11] Wang L., Sun Y. (2022). Image classification using convolutional neural network with wavelet domain inputs. *IET Image Processing*.

[12] Scanlon M. (2018). Semantic annotation of aerial images using deep learning, transfer learning, and synthetic training data. *Thesis for: Masters of Science in Computer Science (Data Analytics), *.

[13] Aldoski J. (2022). *Image Classification Accuracy Assessment*.

[14] Asgher U., Khalil K., Khan M. J. (2020). Enhanced accuracy for multiclass mental workload detection using long short-term memory for brain–computer interface. *Frontiers in Neuroscience*.

[15] Goutte C., Gaussier E. (2005). A probabilistic interpretation of precision, recall and F-score, with implication for evaluation. *Lecture Notes in Computer Science*.

[16] Mahum R., Irtaza A., Nawaz M., Nazir T., Masood M., Mehmood A. (2021). *A Generic Framework for Generation of Summarized Video Clips Using Transfer Learning (SumVClip)*.

[17] Griffin G., Holub A., Perona P. (2007). Caltech-256 Object Category Dataset. http://wwwvisioncaltechedu/Image_Datasets/Caltech256/.

[18] Krizhevsky A. (2009). Learning multiple layers of features from tiny images.

[19] Toledo Y., Almeida T., Bernardini F., Andrade E. (2019). *A Case of Study about Overfitting in Multiclass Classifiers Using Convolutional Neural Networks*.

[20] Cai Q., Li F., Chen Y., Li H., Cao J., Li S. (2021). Label rectification learning through kernel Extreme learning machine. *Wireless Communications and Mobile Computing*.

[21] Kurniawan I., Tarwidi D. (2019). QSAR modeling of PTP1B inhibitor by using Genetic algorithm-Neural network methods. *J. Phys. Conf. Ser*.

[22] Srinivasan K., Garg L., Datta D. (2021). Performance Comparison of deep CNN models for detecting driver’s distraction. *Cmc-Tech Science Press*.

[23] Lo W., Yang X., Wang Y. An Xception convolutional neural network for malware classification with transfer learning.

[24] Liu H., Liu M., Li D., Zheng W., Yin L., Wang R. (2022). Recent advances in pulse-coupled neural networks with applications in image processing. *Electronics*.

[25] Yang Z., Lian J., Guo Y. (2019). An overview of PCNN model’s development and its application in image processing. *Archives of Computational Methods in Engineering*.

[26] Duan X., Gou M., Liu N., Wang W., Qin C. (2020). High-capacity image steganography based on improved Xception. *Sensors*.

[27] Kassani S. H., Kassani P., Khazaeinezhad H. R., Wesolowski M. J., Schneider K., Liu L. Diabetic retinopathy classification using a modified Xception architecture.

[28] Datta D., Mittal D., Mathew N. P., Sairabanu J. Comparison of performance of parallel computation of CPU cores on CNN model.

[29] Qassim H., Verma A., Feinzimer D. Compressed residual-VGG16 CNN model for big data places image recognition.

[30] Bansal M., Kumar M., Sachdeva M., Mittal A. (2021). Transfer learning for image classification using VGG19: Caltech-101 image data set. *Journal of Ambient Intelligence and Humanized Computing*.

[31] Elshennawy N. M., Ibrahim D. M. (2020). Deep-pneumonia framework using deep learning models based on chest X-ray images. *Diagnostics*.

[32] Bhakti B., Shubham Gajre S., Talbar S. Eff-UNet: a novel architecture for semantic segmentation in unstructured environment.

[33] Eckhorn R., Reitboeck H. J., Arndt M., Dicke P. (1990). Feature linking via synchronization among distributed assemblies: simulations of results from cat visual cortex. *Neural Computation*.

[34] Lindblad T., Kinser J. M. (2005). *Image Processing Using Pulse-Coupled Neural Networks*.

[35] Tanaka M., Watanabe T., Baba Y., Kurita T., Mishima T. Autonomous foveating system and integration of the foveated images. *IEEE*.

[36] Canny J. (1986). A computational approach to edge detection. *IEEE Transactions on Pattern Analysis and Machine Intelligence*.

[37] Gedraite E. S., Hadad M. (2011). Investigation on the effect of a Gaussian Blur in image filtering and segmentation. *Proceedings ELMAR-2011*.

[38] Hayashi T., Tsubouchi T. (2022). Estimation and sharpening of blur in degraded images captured by a camera on a moving object. *Sensors*.

[39] Budde L. E., Bulatov D., Iwaszczuk D. (2021). Identification of misclassified pixels in semantic segmentation with uncertainty evaluation. *The International Archives of the Photogrammetry, Remote Sensing and Spatial Information Sciences*.

[40] Ibrahem W. N. (2016). Wavelets and multiresolution processing, *Image processing*. *Lecture*.

[41] Houssein E. H., Ali M. A. S., Hassanien A. E. An image steganography algorithm using haar discrete wavelet transform with advanced encryption system.

[42] Zhang W., Yao G., Yang B., Zheng W., Liu C. (2022). Motion prediction of beating heart using spatio-temporal LSTM. *IEEE Signal Processing Letters*.

[43] Guo F., Yang B., Zheng W., Liu S. (2021). Power frequency estimation using sine filtering of optimal initial phase. *Measurement*.

[44] Xu C., Yang B., Guo F., Zheng W., Poignet P. (2020). Sparse-view CBCT reconstruction via weighted Schatten p-norm minimization. *Optics Express*.

[45] Rezende E., Ruppert G., Carvalho T., Theophilo A., Ramos F., Latifi S. (2018). Malicious software classification using VGG16 deep neural network’s bottleneck features. *Information Technology-New Generations. Advances in Intelligent Systems and Computing*.

[46] Rinky B. P., Mondal P., Manikantan K., Ramachandran S. (2012). DWT based feature extraction using edge tracked scale normalization for enhanced face recognition. *Procedia Technology*.

